# Pluripotency of *Wolbachia* against Arboviruses: the case of yellow fever

**DOI:** 10.12688/gatesopenres.12903.2

**Published:** 2019-04-16

**Authors:** Marcele Neves Rocha, Myrian Morato Duarte, Simone Brutman Mansur, Bianca Daoud Mafra e Silva, Thiago Nunes Pereira, Talita Émile Ribeiro Adelino, Marta Giovanetti, Luis Carlos Junior Alcantara, Franciele Martins Santos, Victor Rodrigues de Melo Costa, Mauro Martins Teixeira, Felipe Campos de Melo Iani, Vivian Vasconcelos Costa, Luciano Andrade Moreira

**Affiliations:** 1 Mosquitos Vetores, IRR, Fundação Oswaldo Cruz, Belo Horizonte, MG, Brazil; 2 Serviço de Virologia e Riquetsioses, Fundação Ezequiel Dias-LACEN, Belo Horizonte, MG, Brazil; 3 Laboratório de Flavivírus, IOC, Fundação Oswaldo Cruz, Rio de Janeiro, RJ, Brazil; 4 Laboratório de Genética Celular e Molecular, Universidade Federal de Minas Gerais, Belo Horizonte, MG, Brazil; 5 Centro de Pesquisa e Desenvolvimento de Fármacos, Universidade Federal de Minas Gerais, Belo Horizonte, MG, Brazil; 6 Research Group in Arboviral Diseases, Universidade Federal de Minas Gerais, Belo Horizonte, MG, Brazil; 7 Immunopharmacology Lab, Universidade Federal de Minas Gerais, Belo Horizonte, MG, Brazil

**Keywords:** Wolbachia, Aedes aegypti, Yellow fever virus, vector competence

## Abstract

**Background**: Yellow fever outbreaks have re-emerged in Brazil during 2016-18, with mortality rates up to 30%. Although urban transmission has not been reported since 1942, the risk of re-urbanization of yellow fever is significant, as *Aedes aegypti* is present in most tropical and sub-tropical cities in the World and still remains the main vector of urban YFV. Although the YFV vaccine is safe and effective, it does not always reach populations at greatest risk of infection and there is an acknowledged global shortage of vaccine supply. The introgression of *Wolbachia* bacteria into *Ae. aegypti* mosquito populations is being trialed in several countries ( www.worldmosquito.org) as a biocontrol method against dengue, Zika and chikungunya. Here, we studied the ability of *Wolbachia* to reduce the transmission potential of *Ae. aegypti* mosquitoes for *Yellow fever virus* (YFV).

**Methods:** Two recently isolated YFV (primate and human) were used to challenge field-derived wild-type and *Wolbachia*-infected ( *w*Mel +) *Ae. aegypti* mosquitoes. The YFV infection status was followed for 7, 14 and 21 days post-oral feeding (dpf). The YFV transmission potential of mosquitoes was evaluated via nano-injection of saliva into uninfected mosquitoes or by inoculation in mice.

**Results:** We found that *Wolbachia* was able to significantly reduce the prevalence of mosquitoes with YFV infected heads and thoraces for both viral isolates. Furthermore, analyses of mosquito saliva, through indirect injection into naïve mosquitoes or via interferon-deficient mouse model, indicated *Wolbachia* was associated with profound reduction in the YFV transmission potential of mosquitoes (14dpf).

**Conclusions:** Our results suggest that *Wolbachia* introgression could be used as a complementary strategy for prevention of urban yellow fever transmission, along with the human vaccination program.

## Introduction

Arboviruses impose a substantial disease burden on the human population ^1, 2^. Most recently, the *Zika virus* re-emerged in 2014, and unexpectedly caused serious congenital infections in pregnant women and Zika fetal syndrome in affected newborns in several American countries in 2016 and 2017 ^3^. *Chikungunya virus* caused massive epidemics in the Americas in 2014 and still circulates in several countries ^4^.

The *Yellow fever virus* (YFV) is a member of the Flaviviridae family and transmitted by sylvan mosquitoes of the genus *Haemagogus* and *Sabethes*, in South America and *Aedes aegypti* in urban settings ^5– 8^. Monkeys are important reservoirs of YFV in sylvan environments. Encroachment by humans into environments where competent mosquito vectors and infected monkeys co-exist is the commonest reason for spillover of YFV transmission to human populations. Although the last reported cases of urban transmission in Brazil occurred in 1942, in 2016–2017, the country faced major outbreaks of the disease mainly in the states of Minas Gerais, Espírito Santo and Rio de Janeiro. In 2018, the epidemic also extended to São Paulo State ^9^. According to the Brazilian Ministry of Health, from July 2017 to April 2018, there were 1,127 YFV cases with 328 deaths, with no evidence of urban transmission. Although the YFV vaccine is safe and effective, it does not always reach populations at greatest risk of infection and there is an acknowledged global shortage of vaccine supply ^10^.

Recent studies have shown that anthropophilic mosquitoes, such as *Aedes aegypti* and *Aedes albopictus,* as well as Brazilian enzootic mosquitoes, such as *Haemagogus leucocelaenus* and *Sabethes albiprivus,* were highly susceptible to American and African YFV strains ^11– 13^. Therefore, the possible resurgence of urban epidemics of YFV in South America has to be constantly monitored by public health authorities ^13^. Population control of *Ae. aegypti* mosquitoes using insecticides has been a mainstay of vector-borne disease control methods for decades but is undermined by widespread insecticide resistance. A promising innovative strategy, based on a bacterium called *Wolbachia pipientis*, has been trialed in many countries. *Wolbachia* is a maternally transmitted bacterial endosymbiont and is naturally present in at least 40% of all insect species ^14^. The World Mosquito Program is deploying *Wolbachia* as a self-sustaining disease control agent on the basis that *Wolbachia* reduces the transmission potential of *Ae. aegypti* mosquitoes for dengue ^15^, Zika ^16^ and chikungunya viruses ^17^.

Here, we studied the ability of *Wolbachia* to suppress YFV infectivity in *Ae. aegypti* mosquitoes. Two virus isolates were used: one from a human clinical sample and another one of primate origin. We found that *Wolbachia* had a major impact on virus replication in mosquitoes, as well as reduced the potential of YFV transmission via saliva, as indirectly determined via mosquitoes or a mouse model.

## Methods

### Sample collection

The first sample named M377_IV|Human|MinasGerais_PadreParaíso|2017-02-04 (YFV377H) was isolated from human serum, positive for YFV by RT-qPCR (CT = 28.95) in February, 2017 from Padre Paraíso city (northeast of Minas Gerais state). The other sample named M127_IV|Primate|MinasGerais_NovaLima|2018-01-15 (YFV127P) was isolated from the liver of a non-human primate found dead in January 2018, in Nova Lima city, in the center-south of Minas Gerais state, positive for YF via RT-qPCR (CT = 17.19). Sequencing of both isolates was performed and is described below. Viral isolation was confirmed by two methodologies: indirect immunofluorescence (IFA) and RT-qPCR. IFA was performed with a monoclonal YFV antibody donated by Evandro Chagas Institute (Arbovirology and Hemorrhagic Fevers Section) and conjugated goat anti-mouse IgG labeled with fluorescein FITC (MP Biomedicals) according to Adungo *et al.* 2016 ^18^ with modifications. Images were obtained using an Olympus microscope model BX51 with DP72 camera and DP-2BSW software. Viral molecular confirmation was performed using RNA extracted from the culture supernatant of each isolate, followed by amplification of the genetic material as described below in the viral detection section according to Domingo *et al.* 2012 ^19^. For mosquito infections, the YFV isolates were replicated in C636 cells ( *Ae. albopictus*) cultured in Leibovitz 15 medium (Gibco) supplemented with 10% fetal bovine serum (FBS) (Gibco) for 5 days at 28°C. Viral load was confirmed by RT-qPCR and later through plaque assays (PFU) in VERO cells (CCL81) grown in DMEM medium (Gibco) and 3% Carboxymethylcellulose (Sigma) supplemented with 2% FBS (Gibco) at 37°C and 5% CO _2_^20^.

### Nucleic acid isolation and virus genome sequencing

Viral RNA was isolated from 200µL of each sample using MagNA Pure 96 (Roche) following manufacturer’s recommendations. To confirm the viral presence in isolates, RT-qPCR was performed, according to Domingo *et al.* 2012 ^19^.

A real-time nanopore sequencing strategy with previously developed primers ^21^, was applied to both RT-qPCR-positive samples. For these samples, extracted RNA was converted to cDNA using GoScript™ Reverse Transcriptase (Promega) and random hexamer priming. Whole-genome amplification by multiplex PCR was attempted using GoTaq® qPCR Master Mix (Promega), the 500bp sequencing primer scheme and 35 cycles using the adapted protocol ^21^. Electrophoresis (2% agarose gel) was used to confirm the expected bands and to purify the specific amplicons using Invitrogen™ E-Gel™ SizeSelect, followed by quantification using fluorimetry with the Qubit dsDNA High Sensitivity assay on the Qubit 3.0 instrument (Life Technologies).

Template was amplified with end point PCR to increase template concentration following the Ion Plus Fragment Library Kit recommendation and PCR products were cleaned-up using AmpureXP purification beads (Beckman Coulter). Emulsion PCR was performed to amplify the library using Ion PGM™ Hi-Q™ View OT2 Kit (Thermo Fisher Scientific) and the Ion OneTouch 2 system (Thermo Fisher Scientific). Ion Sphere particles (ISPs) were enriched using the Ion OneTouch ES (Thermo Fisher Scientific). Enriched ISPs were sequenced using the Ion Torrent Personal Genome Machine (PGM) and the Ion PGM Hi-Q Sequencing kit (Thermo Fisher Scientific), with the Ion 314 chip. All procedures above followed manufacturer’s instructions.

Consensus genome sequences from fastq file were produced by alignment of two-direction reads by using a reference YFV genome. Quality control on raw sequence data have been performed using FastQC ^22^. Bowtie 2 was used for mapping reads to a reference using Galaxy ^23^. Only positions with ≥ 20× genome coverage were used to produce consensus sequences. Regions with lower coverage and those in primer-binding regions were masked with N characters.

In order to identify the origin of the YFV genome from the samples, we performed a maximum likelihood (ML) phylogenetic analysis using the newly two nucleotide sequences recovered in this study plus 125 reference YFV complete genome sequences from each different genotype (South American I n=84; South American II n=2; West African n=23; East African n=16) already published in peer-reviewed journals, for which sampling year and geographic location is available. Full details of the reference sequences used are provided in Extended data: Table S1.

Consensus sequences were aligned using MAFFT v.7 ^24^. Maximum likelihood phylogenetic trees were estimated using IqTree ^25^ under a GTR + Γ _4_ nucleotide substitution model. Statistical support for phylogenetic nodes was estimated using a bootstrap approach (100 replicates).

The phylogenetic signal has been investigated with the likelihood mapping method by analyzing groups of four sequences, randomly chosen, called quartets. Likelihood mapping analyses was performed with the program TREE-PUZZLE by analyzing 10,000 random quartets ^26^.

### Mosquitoes and infections

Wild type *Aedes aegypti* mosquitoes collected in the neighborhood of Urca, Rio de Janeiro-RJ, Brazil in 2018 were reared in the laboratory for five generations and confirmed for the absence of *Wolbachia* (WT). *Wolbachia w*Mel strain-containing mosquitoes ( *w*Mel +) were obtained from the colony maintained by the World Mosquito Program (WMP) Brazil laboratories in Belo Horizonte, which is backcrossed every five generations with Urca male mosquitoes. They were reared in a controlled environment at 27 ± 2°C and 60 ± 10% relative humidity. Four to six days-old female mosquitoes were starved for 20 to 24 hours and subsequently offered YFV virus culture supernatant mixed with washed human red blood cells (RBCs) (2:1 ratio). The viral titer offered to mosquitoes was 4 × 10 ^5^ PFU/mL for YFV377H and 1.4 × 10 ^6^ PFU/mL for YFV127P. RBCs were washed three times for removal of potential YFV vaccine antibodies. Mosquitoes were allowed to feed for one hour and then, engorged females were selected and maintained in triple containment, under BSL-2 conditions. Sucrose solution (10%) was offered *ad libitum* during the extrinsic incubation period. Viral load was analyzed at 7, 14 and 21 days post feeding (dpf), via RT-qPCR and number of mosquitoes analyzed per group were presented in Figures 3B, C and D and ranged from 17 to 20. Additionally, a subset of mosquitoes (at 7dpf) received an extra blood meal and were collected at 14dpf, when *Wolbachia* density and viral load was determined. *Wolbachia* density was analyzed in the three time-points, being 40 mosquitoes at 7dpf, 39 at 14dpf and 38 mosquitoes after 21dpf. The blood used in the infective feedings was obtained from a blood bank (Hemominas) through an agreement signed between both institutions (OF.GPO/CCO-Nr224/16). As a laboratory routine each blood bag is previously tested for dengue, Zika, chikungunya, mayaro and yellow fever, through RT-qPCR to rule out any cross-infection that could interfere with the results.

### Mosquito saliva transmission assays

In order to check the ability of mosquitoes to transmit the virus, saliva samples from infected mosquitoes were individually collected at 14 dpf. After removal of legs and wings, mosquitoes had their proboscis introduced into 10 μL tips, containing 50% Fetal Bovine Serum (FBS) (Gibco) and 30% sugar solution and allowed to salivate for 30 minutes. Mosquitoes and solution containing the saliva were stored at -70°C until RNA extraction of the heads/thoraces and/or nanoinjection of the saliva into naive mosquitoes (WT). Individual saliva samples were injected into WT mosquitoes, after 2 to 4 days of emergence. Each mosquito received 276 nL and were kept for 5 days before whole body RNA extraction, followed by RT-qPCR. Usually, with one saliva sample it is possible to inject 15 mosquitoes, but due mortality, 8 mosquitoes were analyzed from each nanoinjected saliva sample.

*In vivo* experiments were conducted using type I interferon receptor deficient mice (A129 ^−/−^), SV129 background. A129 ^-/- ^originally from *The Jackson Laboratories* (reference 010830) were obtained from Biotério de Matrizes da Universidade de São Paulo (USP) and kept under specific pathogen-free conditions at Immunopharmacology Lab at UFMG. Mice were housed in filtered-cages of 28x13x16 cm with autoclaved food and water available ad libitum on ventilated shelves (Alesco). A maximum of 4 mice were kept per cage. Mice were housed under standard conditions with controlled temperature (18–23 degrees) humidity (40–60%) and 12/12h dark light cycle. Sample sizes for *in vivo* studies were determined using the G*Power 3.1 software package. In each experiment we used 4 mice on YFV377H or YFV127P groups and 6 mice per group on saliva YFV 377H or 127P infected mosquitoes (WT or *w*Mel+) groups. Mice from the same litter were added to either mock- or YFV infected groups, or test or control groups as appropriate. No randomization protocol was utilized. For most of the experiments, no blinding was involved except for body weight and hind paw swelling analysis. Bioanalysis from viral loads and cell count assay experiments was blinded. Groups were divided by codenames on the day of euthanasia. Different researchers performed the euthanasia or analyzed the data. Each experiment was replicated twice and all attempts at replication were successful. For the experiments, adult A129 ^-/-^ mice (7 to 9 weeks old, 20-22g) were inoculated with 1 × 10 ^4^ PFU with either YFV377H or YFV127P viruses’ strains or with a pool of saliva samples (n=2) either from the WT or *w*Mel+ groups via subcutaneous (intraplantar) route/50μl paw (right hind paw). Morbidity parameters such as body weight loss, total and differential counts of blood leukocytes and paw edema were evaluated daily. Total cell counts were carried out in Trypan blue-stained cells in a Neubauer chamber and differential cell counts on blood smears stained with May-Grunwald-Giemsa using standard morphological criteria. Paw edema was assessed by measuring paw swelling using a pachymeter. Finally, viable viral loads and viral RNA were analyzed in plasma and different tissues of mice upon saliva inoculation, as shown below.

All animal experiments involving YFV infection and *Wolbachia* saliva inoculation were conducted following the ethical and animal welfare regulations of the Brazilian Government (law 11794/2008). The experimental protocol was approved by the Committee on Animal Ethics of the Universidade Federal de Minas Gerais (CEUA/UFMG, permit protocol no. 84/2018). All surgeries were performed under ketamine/xylazine anesthesia and all efforts were made to minimize animal suffering. Studies with YFV were conducted under biosafety level 2 (BSL-2) containment at Immunopharmacology Lab from Instituto de Ciências Biológicas (ICB) at Universidade Federal de Minas Gerais.

### Viral detection in mosquitoes and mice

Detection of viral RNA on infected mosquitoes and mice samples were performed through quantitative real-time PCR (RT-qPCR) using LightCycler® Multiplex RNA Virus Master (Roche), according to the previously published protocol ^27^. RNA extractions were performed following manufacturer's protocols. Mosquito samples were processed through the High Pure Viral Nucleic Acid kit (Roche), mice tissue samples (liver, spleen) were extracted with Trizol (Invitrogen), whereas mice lymph node samples were isolated with the QIAamp® Viral RNA kit (Qiagen). Multiplex reactions were performed with primers and probes described in Table 1. Reactions were performed on a Lightcycler96 real-time PCR machine (Roche) with the following program: first step at 50°C for 10 min for reverse transcription, 95°C for 30 sec for inactivation and initial denaturation and 95°C for 5 sec followed by 60°C for 30 sec for 40 cycles. The reaction volume was 10 μL (5× RT-PCR Reaction Mix (Roche), 200× RT-enzyme solution (Roche), 2.5 μM each primer (IDT) and 2 μM YF (target yellow fever) probe (IDT) and 1 μM WSPTM2 (target *w*Mel-specific) probe and 0.7 μM RPS 17S (target *Ae. aegypti* ribosomal S17) probe. For mouse samples, only the YFV probe was used. A fraction (1/20) of the total isolated RNA was used in the reactions. Head and thorax samples from YFV-challenged mosquitoes were analyzed in duplicate through RT-qPCR and viral and *Wolbachia* quantification were performed in comparison with serial dilution of a standard curve of the respective genes cloned into the pGEMT plasmid (Promega) ^16, 27^. Therefore, it was possible to calculate the number of copies per tissue. As a mosquito control gene, we used the RPS 17S sequence of *Ae. aegypti* (Moreira 2009) ^15^. Viable viral loads were quantified by titration assay in permissive Vero cells as described in Costa *et al.,* 2012 ^29^.

**Table 1.** Sequence of primers and probes used in this study.

	Sequence 5’→3’	Reference
YFV Forward	GCTAATTGAGGTGYATTGGTCTGC	19
YFV Reverse	CTGCTAATCGCTCAAMGAACG	
YFV Probe	**FAM**/ATCGAGTTG/ **ZEN**/CTAGGCAATAAACAC/ **3lABkFQ**	
WSPTM2 Forward	CATTGGTGTTGGTGTTGGTG	15
WSPTM2 Reverse	ACACCAGCTTTTACTTGACCAG	
WSPTM2 Probe	**CY5**/TCCTTTGGA/ **TAO**/ACCCGCTGTGAATGA/ **3lAbRQSp**	
RPS17 S Forward	TCCGTGGTATCTCCATCAAGCT	28
RPS 17S Reverse	CACTTCCGGCACGTAGTTGTC	
RPS17 S Probe	**HEX**/CAGGAGGAG/ **ZEN**/GAACGTGAGCGCAG/ **3lABkFQ**	

### Statistical analysis

All statistical analyses were performed on Prism (Graphpad Version 7.04). Initially the D'Agostino and Person normality test was performed. *Wolbachia* density data as well as viral load were compared using the non-parametric Mann-Whitney test. Statistical analyzes for the mouse data were performed with ANOVA one-way test. The significance level was set for *p* values less than 0.05.

## Results

### Viral isolation and sequencing

**Figure 1.**
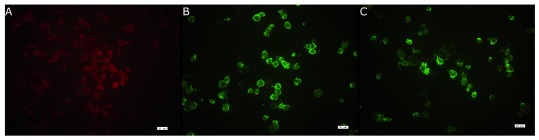
*Yellow fever virus* (YFV) immunofluorescence in C636 cells. ( **A**) Control cells without virus, ( **B**) cells infected with YFV 377 H and ( **C**) cells with YFV127 P. Green fluorescence depicts YFV in cells marked with a monoclonal YFV antibody conjugated goat anti-mouse IgG labeled with fluorescein FITC.

**Figure 2.**
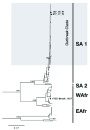
Maximum likelihood phylogeny obtained using two novel complete *Yellow fever virus* sequences plus 126 YFV reference sequences from each different genotype (South American I; South American II; West African; East African). ML showing the two newly genomes belongs to South American I (SA1) genotype. SA2, WAfr, and EAfr indicate the South America II, West Africa, and East Africa genotypes, respectively. The scale bar is in units of substitutions per site (s/s). Node labels indicate bootstrap support values.17DD, the vaccine strain used in Brazil.

Two plasma samples (one human and one from a non-human primate) were isolated from the diagnostic service of Fundação Ezequiel Dias, the State Reference Laboratory of Minas Gerais, Brazil. Viral isolation was confirmed by indirect immunofluorescence (IFA), showing the typical signal of fluorescence for both isolates ( Figure 1B and C). Both samples were successfully sequenced with PGM (Personal Genome Machine) technology with adapted overlapping multiplex PCR protocol, as shown in Table 2. The phylogenetic analysis showed that the isolates obtained from the two samples (M377_IV and M127_IV) belonged to the South American genotype I and clustered closely with strong bootstrap support (>90%) with the recent sequences, isolated in Minas Gerais, from the current outbreak ( Figure 2) ^30^.

**Table 2.** Main results obtained by sequencing.

Sample ID	Acession number (GenBank)	CT value	Coverage	Mean deph	N° of reads	Mapped reads	Mean mapping quality
M377_IV	MK249065	13.82	92.5%	4,004 X	218,811	216.613 (99%)	37
M127_IV	MK249066	16.68	93%	6,640 X	361,806	358.522 (99%)	37.02

### *Wolbachia* density

**Figure 3.**
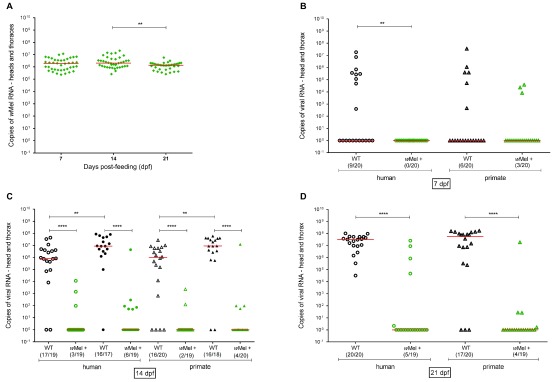
Interference of *Wolbachia* towards *Yellow fever virus* and *Wolbachia* absolute quantification. Wild type (WT) or positive ( *w*Mel +) were orally infected with two YFV isolates and virus dissemination in mosquitoes was analyzed at different times post infection. ( **A**) YFV infected mosquitoes’ heads and thoraces were analyzed for *Wolbachia* density at different times post-infection through real time RT-qPCR, based on a *Wolbachia* standard curve. Red lines indicate the median *w*Mel copies (Mann-Whitney U test, ** *p*=0.0062). ( **B**) Analysis of copies of viral RNA on 7dpf -WT x *w*Mel+ (** *p*=0.0028) and YFV Human x Primate WT ( *p* = 0.43), ( **C**) 14dpf YFV Human and Primate WT x *w*Mel + (**** *p*<0.0001), YFV Human x Primate WT ( *p*=0.75), YFV Human x Primate extra blood meal WT ( *p*=0.78), YFV Human WT x WT extra blood meal (** *p*=0.0061) and YFV Primate WT x WT extra blood meal (** *p*= 0.0056) and ( **D**) 21 dpf -WT x *w*Mel+ (**** *p*=0.0001) and YFV Human x Primate ( *p*= 0.51). Empty black circles and triangles are WT mosquitoes, whereas empty green circles and triangles depict mosquitoes with *w*Mel +. Black filled circles and triangles are mosquitoes that received a second blood meal. The red line indicates the median YFV copies.

Absolute quantification of *Wolbachia* in mosquitoes were analyzed in the heads and thoraces of *Wolbachia*-positive mosquitoes ( *w*Mel +) after challenge with YFV. There was no difference in *Wolbachia* density among heads and thoraces, collected at 7 or 14 days post feeding (dpf), as shown in Figure 3A. However, *Wolbachia* density presented a slight reduction at 21dpf, which was statistically significant in relation to 14dpf ( *p* = 0.0062, Mann Whitney). The median at 14dpf was 2.04 × 10 ^6^ copies per head/thorax whereas at 21dpf, it decreased to 1.37 × 10 ^6^.

### *Wolbachia* reduces susceptibility of *Ae. aegypti* to YFV infection

In mosquitoes without *Wolbachia* (WT) the prevalence of YFV infection of heads and thoraces was 30–45% at 7dpf, and 80–89% at 14dpf. For those mosquitoes that received a 2 ^nd^ blood meal, the prevalence was 89 to 94% at 14dpf and 85 to 100% at 21dpf. There was no significant difference between infection rates resulting from the human or primate virus isolates ( Figure 3). In heads and thoraces of *Wolbachia*-positive mosquitoes ( *w*Mel +) the infection rate ranged from 0 to 15% at 7dpf, 11 to 16% at 14dpf, 20 to 32% at 14dpf when mosquitoes received a second blood meal, and 20 to 25% at 21dpf ( Figure 3). Again, there was no major difference between viral isolates.

The infection rate observed at 7dpf was low for both viral isolates ( Figure 3B). At day 7, the presence of *Wolbachia* was already associated with a marked decrease in viral titers in mosquitoes ( Figure 3B). At 14dpf, there was a significant increase in the number of viral copies in WT mosquitoes ( Figure 3C). Further increase on viral load was observed when mosquitoes received a second blood meal 7 days after the infective meal and were analyzed at 14 dpf. This increase was statistically significant for both isolates ( *p* <0.01, Mann Whitney). This may have been due to the fact that the second blood supplied extra important nutrients for viral replication. At 21dpf, the infection reached 100% for the human isolate with a median of 3.15 × 10 ^7^ viral copies. For the primate isolate, although the infection rate was lower (85%), the viral load was higher with a median of 5.61 × 10 ^7^ viral copies per head/thoraces. Regardless of the strain of virus used, viral loads were remarkable lower in presence of *Wolbachia* at all time points ( Figure 3B–D). In addition, there was no increase in viral load in *w*Mel + mosquitoes after supplying a second blood meal ( Figure 3C).

### Virus transmission through saliva

**Figure 4.**
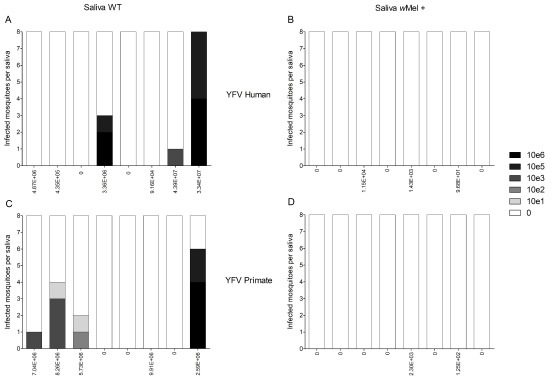
Indirect evaluation of yellow fever virus (YFV) transmission through mosquito saliva. Saliva from both groups of infected mosquitoes were collected at 14 dpf. Individual saliva samples (WT or *w*Mel +) were analyzed into eight naïve (WT) mosquitoes (bars) and, after five days, these injected mosquitoes were analyzed. ( **A**) Mosquitoes injected with mosquito saliva or ( **B**) *w*Mel+ mosquitoes, challenged with human virus. ( **C**) Mosquitoes injected with WT mosquito saliva or ( **D**) *w*Mel+ mosquitoes, challenged with primate virus. Values below each bar depicts the viral load of each mosquito head and thorax which donated that saliva. Positive mosquitoes were quantified through RT-qPCR and the grey-scale represents the number of YFV copies (0 to 10 ^6^ copies), per mosquito.

Next, we evaluated the ability of orally infected mosquitoes to transmit the virus. We first collected saliva from infected mosquitoes at 14 dpf, from both groups of mosquitoes and virus isolates. We then injected a number of saliva samples into eight naïve (WT) mosquitoes and, after five days, we checked whether those mosquitoes became infected through RT-qPCR, demonstrating that a particular saliva was infectious. As shown in Figure 4, when saliva samples originated from *w*Mel + mosquitoes, no mosquitoes became infected. This assay shows, indirectly, the potential of *Wolbachia* to completely abrogate YFV transmission potential of *Ae. aegypti* mosquitoes. Nevertheless, saliva originating from WT mosquitoes was able to infect 20% of the naïve-injected mosquitoes.

**Figure 5.**
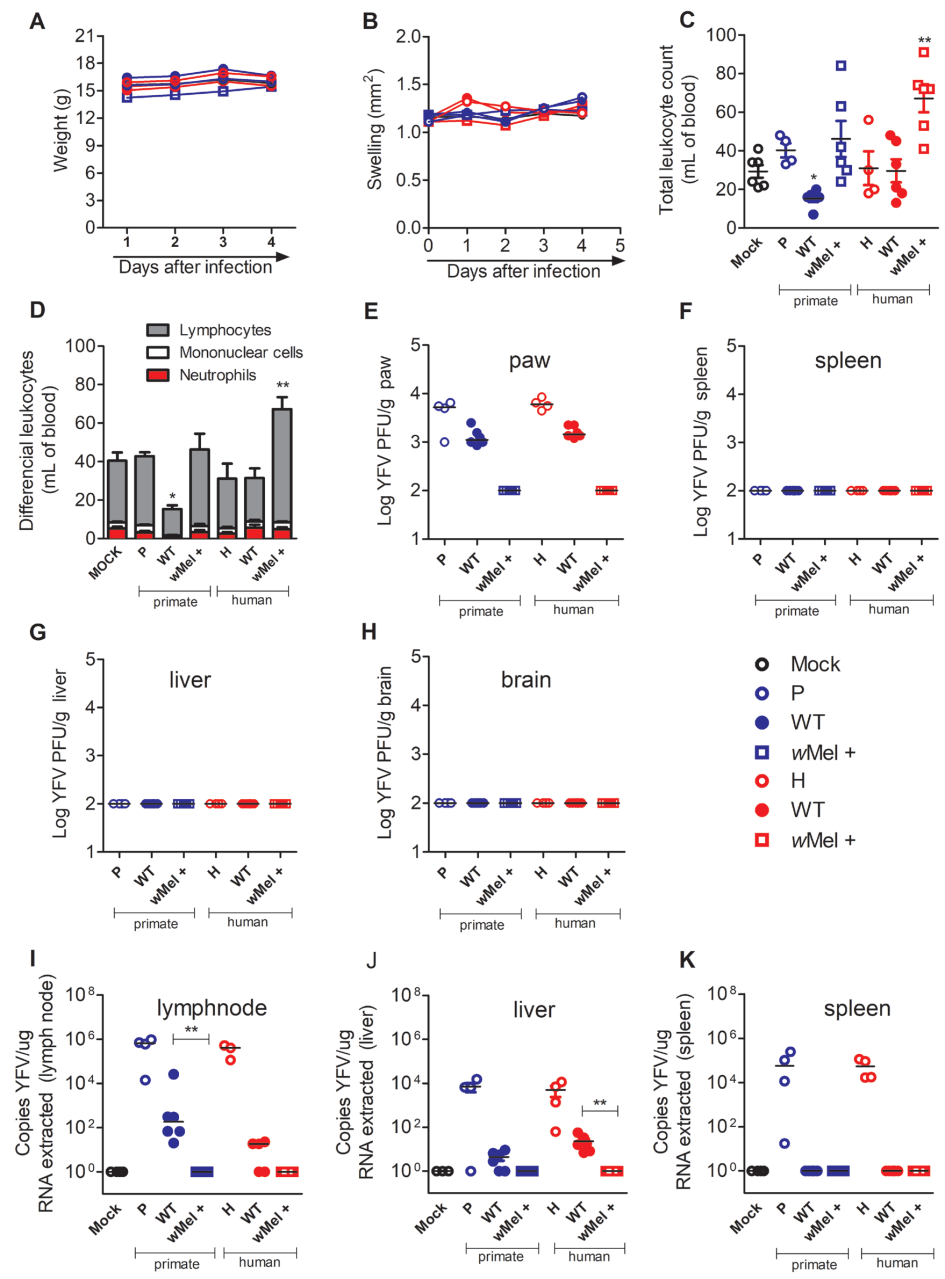
Saliva from *Wolbachia*-positive mosquitoes lose its capacity to transmit yellow fever virus *in vivo*. A129 ^-/- ^mice were inoculated with 1 × 10 ^4 ^PFU of YFV primate (empty blue circles) and human YFV (empty red circles) or with a pool of saliva from wild tipe (WT) YFV primate (full blue circles), WT YFV human (full red circles), *Wolbachia*-positive ( *w*Mel +) YFV primate (empty blue squares) and *Wolbachia*-positive YFV human (empty red squares) previously infected with YFV via intraplantar route (50 μl/paw). Control mice (MOCK group) received 50 μl of PBS solution (empty black circle). ( **A**) Body weight analysis shown as body weight (g) of mice. ( **B**) Paw volume measured daily and shown as swelling (mm ^2^). On day 4 post-infection mice were euthanized and the following analysis performed. ( **C–D**) Total and differential leukocyte counts in the blood. ( **E–H**) Viable viral loads recovered from paw ( **E**), spleen ( **F**), liver ( **G**) and brain ( **H**) by plaque assay in Vero cells. Results are shown as Log PFU/g of tissue. ( **I–K**) Viral RNA copies recovered from popliteal lymph node ( **I**), liver ( **J**) and spleen ( **K**) by RT-qPCR. Data was presented as mean±SEM or median (n=4 mice for MOCK, n=6 mice for WT P, *w*Mel + P, WT H and *w*Mel + H groups and n=4 for YFV P and YFV H, one-way anova).

Similar experiments were performed by injecting saliva samples from either the WT or *w*Mel + groups into 4-week-old A129 ^-/- ^mice, which are susceptible to arboviral infections ^31, 32^. Results showed that there was no major impact on clinical and laboratory parameters, which is consistent with the relatively low number of viable virus injected ( Figure 5A–D). However, there were viable viruses, as assessed by plaque assay, recovered from the paw of mice inoculated with saliva from WT mosquitoes. Indeed, there was culturable virus when both P (primate) and H (human) strains were used. In contrast, none of the samples from the *w*Mel + groups were positive on the plaque assay ( Figure 5E–H). Consistently with the mosquito saliva findings above, there were higher number of viral RNA copies in draining lymphnode and liver from mice injected with WT saliva than mice inoculated with *w*Mel + saliva ( Figure 5 I–K). Virus isolated from the primate (YFV127P) showed greater presence in liver while the human strain (YFV377H) was more localized at the lymphoid tissue ( Figure 5).

Collectively these results suggest that *Wolbachia*-positive mosquitoes can efficiently suppress YFV replication and reduce virus transmission through saliva.

## Discussion

The ability of *Wolbachia* to reduce the susceptibility of *Ae. aegypti* to disseminated arbovirus infection has been repeatedly demonstrated for dengue ^15^, Zika ^16^, chikungunya ^17^, West Nile ^33^ and mayaro viruses ^27^. We have shown that *w*Mel was able to significantly reduce the infectivity of YFV to mosquitoes, independently of the source of the virus (both human and primate). Previously, it has been shown that two strains of *Wolbachia* ( *w*MelPop and *w*Mel) were able to significantly reduce YFV mosquito infection, although with virus isolated from human cases from Nigeria and Bolivia, in 1987 and 1999, respectively ^34^. Here we evaluated the effect of *Wolbachia* ( *w*Mel strain) towards two recently isolated *Yellow fever virus*, originating from the 2017–2018 outbreaks in Brazil. The *Yellow fever virus* isolates used here have different origins, one originating from a non-human primate found in the city of Nova Lima and another originated from a human case in the city of Padre Paraíso, both in the state of Minas Gerais. Although these cities are located more than 500 km apart, they belong to the same genotype. Besides working with recently isolated virus from human and primate sources, the difference in the present study refers to the way in which this population of mosquitoes have been infected. Furthermore, this study was performed with orally infected mosquitoes, which is closer to natural conditions, in comparison to the previous study which infected mosquitoes through thorax injection, in order to improve mosquito infection ^35^.

The use of *Wolbachia* as an arbovirus control strategy has been developed by the not-for-profit initiative, the World Mosquito Program. The approach offers the prospect of a natural and sustainable method for arbovirus control ^35– 38^. The impact towards reduction of arbovirus has been analyzed ^39, 40^ and early indication of positive effect has been recently reported ^41^. In Brazil, WMP is expanding its coverage into Rio de Janeiro and Niterói municipalities and epidemiological studies in order to determine arbovirus reduction is underway.

The blocking ability conferred by *Wolbachia* has been directly related to the density of the bacterium within main mosquito tissues such as midgut and/or salivary glands ^15, 42^, where viruses replicate to further produce infectious particles ^43^. In our study, and as observed by Pereira *et al.,* 2018 ^27^, the density of *Wolbachia* was constant at 7 or 14 days after virus exposure. However, there was a reduction of *w*Mel + density at 21dpf, which did not impact the blocking ability towards the virus ( Figure 3). The variation on the density (or titer) of *Wolbachia* within the host has been previously observed, which could be related to the aging of the host ^42^.

In the present study, the presence of *Wolbachia* in mosquitoes greatly reduced YFV infection, except for 7dpf, when the infection rate was low in all groups. Further effect of *Wolbachia* towards YFV was verified when individually collected mosquito saliva was injected into naïve mosquitoes or into a susceptible mice strain and their infectivity was analyzed. This first technique has been widely used by our group and others ^16, 27, 44^, and it is a robust proxy of the potential of individual saliva towards virus transmission. When the source of saliva came from *Wolbachia*-positive mosquitoes, there was no infection in any injected mosquito. Through projection of these results into natural conditions, the YFV transmission could be greatly reduced, as previously modeled for *Dengue virus* ^39^.

Another interesting fact of this work was the increase in viral load observed after the second blood feeding in WT mosquitoes. This same fact was not observed in *w*Mel + mosquitoes. This shows that the blocking ability of *Wolbachia* persists even after the addition of extra blood nutrients (through a second blood meal) and that its blocking effect occurs within 7 days after infection. The reason to include the second blood meal was that antibodies to YFV could be present in the blood and therefore, promoting negative effect towards the virus in WT mosquitoes, but this was not the case. Caragata *et al.* (2013) ^45^ studied the effect of cholesterol towards the *Drosophila C virus*. This mechanism could be present in our experimental mosquitoes, but further studies on this aspect should be developed.

Interestingly, in our experiments, the overall infectivity in mosquitoes was not high, even in control (no *Wolbachia*) mosquitoes. This shows the reduced vector competence of natural Brazilian *Ae. aegypti* populations, which could explain why most of the cases reported on the recent outbreaks in Brazil were in proximity to green areas of parks and forests, where natural YFV mosquito vectors such as *Haemagogus* and *Sabethes* are easily found ^11, 12, 46^.

Our results show that the presence of *w*Mel strain of *Wolbachia* in mosquitoes has the potential to greatly reduce the transmission potential of *Ae. aegypti* for YFV. It is important for public health agencies of arbovirus endemic countries to have constant awareness of the potential of *Ae. aegypti* to become an urban vector for *Yellow fever virus* once again ^6, 47^. If that becomes reality, *Wolbachia*-infected mosquitoes could be a powerful tool for YFV control, along with the currently applied vaccination program ^10, 48^.

Lastly, it is important to consider the possible vector competence of other mosquito species and the possibility of *Wolbachia* virus evolution and, therefore possible lack of interference in this system. If that is the case, other strategies should be consider, as the use of other strains of *Wolbachia* to try to block virus transmission by that particular mosquito species. Integration of complementary strategies are the best solution for arbovirus control.

## Data availability

### Underlying data

The data underlying Figure 3, Figure 4 and Figure 5, as well as viral sequencing data is available from Open Science Framework, https://doi.org/10.17605/OSF.IO/PUZ69 ^49^.

Data are available under the terms of the Creative Commons Zero “No rights reserved” data waiver (CC0 1.0 Public domain dedication).

Genome sequences generated in this study are publicly available in GenBank database: M377_IV|Human|MinasGerais_PadreParaiso|2017-02-04: accession number, MK249065; M127_IV|Primate|MinasGerais_NovaLima|2018-01-15: accession number, MK249066.

### Extended data

**Table S1.** YFV reference strains information, https://doi.org/10.17605/OSF.IO/PUZ69
