# Supplementary material for: Pluripotency of Wolbachia against Arboviruses: the case of yellow fever
Source: Gates Open Res. 2019 Apr 16;3:161. Originally published 2019 Feb 12. [Version 2] doi: 10.12688/gatesopenres.12903.2 (PMC6561079; doi:10.12688/gatesopenres.12903.2)
Supplement: Peer review history

# Peer review: Reviewer response for version 2

**DOI:** 10.21956/gatesopenres.14075.r27067

**Published:** 2019-04-23

**Authors:** Andrew van den Hurk

**Affiliations:** 1 School of Chemistry and Molecular Biosciences, University of Queensland, St. Lucia, Queensland, Australia

The authors have done a good job of addressing the reviewer's comments. I have no further comments to make.

I have read this submission. I believe that I have an appropriate level of expertise to confirm that it is of an acceptable scientific standard.

# Peer review: Reviewer response for version 2

**DOI:** 10.21956/gatesopenres.14075.r27068

**Published:** 2019-04-17

**Authors:** Jayme A. Souza-Neto, Bianca C. Carlos

**Affiliations:** 1 School of Agricultural Sciences, Department of Bioprocesses and Biotechnology, Multiuser Central Laboratory, São Paulo State University (UNESP), Botucatu, Brazil; 2 School of Agricultural Sciences, Department of Bioprocesses and Biotechnology, Multiuser Central Laboratory, São Paulo State University (UNESP), Botucatu, Brazil

In this revised version the authors have fully addressed the points and concerns raised on Version 1.

We have read this submission. We believe that we have an appropriate level of expertise to confirm that it is of an acceptable scientific standard.

# Peer review: Reviewer response for version 1

**DOI:** 10.21956/gatesopenres.13999.r26932

**Published:** 2019-03-25

**Authors:** Jayme A. Souza-Neto, Bianca C. Carlos

**Affiliations:** 1 School of Agricultural Sciences, Department of Bioprocesses and Biotechnology, Multiuser Central Laboratory, São Paulo State University (UNESP), Botucatu, Brazil; 2 School of Agricultural Sciences, Department of Bioprocesses and Biotechnology, Multiuser Central Laboratory, São Paulo State University (UNESP), Botucatu, Brazil

This a very elegant manuscript provided by Rocha and colleagues reporting the effect Wolbachia on yellow fever virus (YFV) transmission by *Aedes aegypti*. For testing this, the authors use an already well-known and -established *Ae. aegypti *line carrying Wolbachia (wMel mosquitoes) to evaluate its competence for two closely related YFV isolates, one from humans and another from primates. Finally, the efficacy of the wMel *Ae. aegypti *strain to block YFV transmission is tested by assessing the number of infectious viral particles in the mosquito saliva by two independent methods: 1) intrathoracic inoculation of mosquitoes or 2) paw inoculation of immune-deficient mice with the saliva of either WT or wMel YFV-infected mosquitoes. Clearly, Wolbachia has a potent effect against YFV, reducing both the infection level of YFV in the mosquito body and the rate of YFV-infected mosquitoes when comparing wMel mosquitoes orally exposed to YFV to their respective WT control mosquitoes. More strikingly, the wMel strain is shown to be unable to transmit YFV by the two indirect transmission assays carried out by the authors. Suggestions and comments are presented below with the intention to improve the manuscript, especially the clarity and accuracy of the methods/design and data presentation. 

  

1. Methods/design description **:** some elements related to this part of the manuscript should be further expanded and detailed for a better understanding and accuracy of the manuscript. Especially, the authors should consider:
  - Improving the description of the YFV infection assays in mosquitoes. For example, it is not clear the number of mosquitoes and replicates used to determine the viral load in the body of WT or wMel mosquitoes. 
  - Similarly, it is important to clarify the design and data presentation of the transmission assays in mosquitoes. For example, it is not clear if each bar on Figure 4 represents the data combination of 8 mosquitoes injected with the same saliva of a given mosquito (1 x 8 x 8) or if it is the data collected from an individual mosquito injected with the saliva of a given individual mosquito (1 x 1 x 8). This may also cause confusion when interpreting this figure as the Y-axis labels refer to “infected mosquitoes per saliva” while the subtitles refer to “the number of YFV copies…per mosquito”. Additionally, the authors should check the position of the graphs on the right (A and C) and left (B and D) panels as they seem not to match their respective description in the subtitles.
  - An important question that was raised is why the authors have chosen to inject mosquito saliva into mice instead of feeding such mosquitoes on mice in order to test transmission directly.
2. Results/hypothesis/conclusions:
  - The leukocyte counts are significantly high in mice inoculated with the saliva of wMel mosquitoes orally exposed to YFV (Figures 5C and 5D). Do the authors have any hypothesis to explain why this is happening?
  - Because the WT mosquito population used in this work presented a relatively low vector competence to YFV, on page 10 (Discussion, second column) the authors infer this phenotype as a representation of Brazilian *Ae. aegypti *populations. While this is a plausible hypothesis, I would suggest the authors to be more cautious with this statement as vector competence to YFV of many other *Ae. aegypti *populations must be tested before one assumes this fact.

We have read this submission. We believe that we have an appropriate level of expertise to confirm that it is of an acceptable scientific standard.

# Response

**Published:** 2019-04-08

**Authors:** Luciano Moreira

**Affiliations:** FIOCRUZ, Brazil

Reviewer – Dr. Jaime A. Souza-Neto and Bianca C. Carlos

This a very elegant manuscript provided by Rocha and colleagues reporting the effect Wolbachia on yellow fever virus (YFV) transmission by *Aedes aegypti*. For testing this, the authors use an already well-known and -established *Ae. aegypti *line carrying Wolbachia (wMel mosquitoes) to evaluate its competence for two closely related YFV isolates, one from humans and another from primates. Finally, the efficacy of the wMel *Ae. aegypti *strain to block YFV transmission is tested by assessing the number of infectious viral particles in the mosquito saliva by two independent methods: 1) intrathoracic inoculation of mosquitoes or 2) paw inoculation of immune-deficient mice with the saliva of either WT or wMel YFV-infected mosquitoes. Clearly, Wolbachia has a potent effect against YFV, reducing both the infection level of YFV in the mosquito body and the rate of YFV-infected mosquitoes when comparing wMel mosquitoes orally exposed to YFV to their respective WT control mosquitoes. More strikingly, the wMel strain is shown to be unable to transmit YFV by the two indirect transmission assays carried out by the authors. Suggestions and comments are presented below with the intention to improve the manuscript, especially the clarity and accuracy of the methods/design and data presentation. 

    1.    Methods/design description **:**some elements related to this part of the manuscript should be further expanded and detailed for a better understanding and accuracy of the manuscript. Especially, the authors should consider:

- Improving the description of the YFV infection assays in mosquitoes. For example, it is not clear the number of mosquitoes and replicates used to determine the viral load in the body of WT or wMel mosquitoes. 

*>> In the section on methods in the item mosquitoes and infection, the requested information was included:*

Viral load was analyzed at 7, 14 and 21 days post feeding (dpf), via RT-qPCR and the number of mosquitoes analyzed per group are presented in figures 3B, C and D, ranging from 17 to 20. Additionally, a subset of mosquitoes (at 7dpf) received an extra blood meal and were collected at 14dpf, when *Wolbachia* density and viral load was determined. *Wolbachia* density was analyzed on mosquitoes from the three time-points as follows: 40 mosquitoes on 7 dpf, 39 mosquitoes on 14 dpf and 38 mosquitoes after 21 dpf.

- Similarly, it is important to clarify the design and data presentation of the transmission assays in mosquitoes. For example, it is not clear if each bar on Figure 4 represents the data combination of 8 mosquitoes injected with the same saliva of a given mosquito (1 x 8 x 8) or if it is the data collected from an individual mosquito injected with the saliva of a given individual mosquito (1 x 1 x 8). This may also cause confusion when interpreting this figure as the Y-axis labels refer to “infected mosquitoes per saliva” while the subtitles refer to “the number of YFV copies…per mosquito”. Additionally, the authors should check the position of the graphs on the right (A and C) and left (B and D) panels as they seem not to match their respective description in the subtitles.

*>> The information requested on the number of nanoinjected mosquitoes was introduced in the methods section (Mosquito saliva transmission assay) as shown below:*

Each mosquito received 276 nL and were kept for 5 days before whole body RNA extraction, followed by RT-qPCR. Usually, with one saliva sample it is possible to inject 15 mosquitoes, but due to mortality, 8 mosquitoes were analyzed from each nanoinjected saliva sample.

*>> The panels were indeed misplaced. Thank you for pointing this out. Please see below:*

Saliva from both groups of infected mosquitoes were collected at 14 dpf. Individual saliva samples (WT or *w*Mel +) were analyzed into eight naïve (WT) mosquitoes (bars) and, after five days, these injected mosquitoes were analyzed. ( **A**) Mosquitoes injected with WT mosquito saliva or ( **B**) *w*Mel+ mosquitoes, challenged with human virus. ( **C**) Mosquitoes injected with WT mosquito saliva or ( **D**) *w*Mel+ mosquitoes, challenged with primate virus. Values below each bar depicts the viral load of each mosquito head and thorax which donated that saliva. Positive mosquitoes were quantified through RT-qPCR and the grey-scale represents the number of YFV copies (0 to 10 ^6^ copies), per mosquito.

- An important question that was raised is why the authors have chosen to inject mosquito saliva into mice instead of feeding such mosquitoes on mice in order to test transmission directly.

*>> The reason why we have not performed the experiment by feeding infected mosquitoes directly on the mice was because we had no biosafety approval to perform these experiments. Therefore, the saliva samples had to be transported to another institution (UFMG), where the mice were located, and then used there.*

    2.     Results/hypothesis/conclusions:- The leukocyte counts are significantly high in mice inoculated with the saliva of wMel mosquitoes orally exposed to YFV (Figures 5C and 5D). Do the authors have any hypothesis to explain why this is happening?**

*>> Mosquito saliva is a very complex concoction of mixture of proteins (>100 proteins), which exerts several functions in the host by circumventing, for example, vasoconstriction, platelet aggregation, coagulation, and inflammation or host hemostasis. Several works in literature have shown that mosquito saliva by itself exerts profound effects on mouse and human immune systems. For example, Vogt and colleagues (2018), using a humanized mice model, have shown that mosquito saliva alters several human blood leukocytes populations such as hematopoietic, NK, NKT, B and myeloid cells. However, the isolate and specific effect of mosquito saliva in modulating blood leukocyte counts was not observed in our work. This finding leads us to believe that wolbachia infection could be associated with such blood leukocyte counts increase. However, when we look closely at the results, we observe that the increase in blood leukocyte counts in mice that received saliva of wMel mosquitoes occurred especially (statistically significant) only upon exposure to human YFV isolate but not after the primate YFV strain inoculation (Fig 5C, 5D). These results suggest that the leukocyte increase observed in the wMel hYFV group was probably due to an interaction between the wolbachia-infected saliva and the human viral isolate in comparison to mice that received the YFV isolate from primates. However, the mechanisms underlying these findings require further investigation.*

*Vogt MB, Lahon A, Arya RP, Kneubehl AR, Spencer Clinton JL, Paust S, Rico-Hesse R.* *Mosquito* *saliva* *alone has profound effects on the human immune system.* *PLoS Negl Trop Dis. 2018 May 17;12(5):e0006439. doi: 10.1371/journal.pntd.0006439.*

- Because the WT mosquito population used in this work presented a relatively low vector competence to YFV, on page 10 (Discussion, second column) the authors infer this phenotype as a representation of Brazilian *Ae. aegypti *populations. While this is a plausible hypothesis, I would suggest the authors to be more cautious with this statement as vector competence to YFV of many other *Ae. aegypti *populations must be tested before one assumes this fact.

*>> Thanks for this point. It was included with the information that the results were obtained in this particular population.*

Besides working with recently isolated virus from human and primate sources, the difference in the present study refers to the way in which this particular population of mosquitoes have been infected.

# Peer review: Reviewer response for version 1

**DOI:** 10.21956/gatesopenres.13999.r26939

**Published:** 2019-03-01

**Authors:** Andrew van den Hurk

**Affiliations:** 1 School of Chemistry and Molecular Biosciences, University of Queensland, St. Lucia, Queensland, Australia

The deployment of *Aedes aegypti* transinfected with the *Wolbachia* is a highly promising strategy for suppressing the transmission of a number of globally important arboviruses, such as dengue, Zika, chikungunya and yellow fever viruses. In the current manuscript, the authors conducted laboratory-based experiments to assess the ability of the *w*Mel strain of *Wolbachia* to inhibit the transmission potential of two outbreak strains of yellow fever virus by *Aedes aegypti*. As has been demonstrated with other flaviviruses in numerous similar studies, *w*Mel inhibited replication and subsequent transmission when compared with *Wolbachia*-negative mosquitoes. This is a relatively well written manuscript describing experiments with outcomes of interest to readers of Gates Open Research. However, there are some components of the manuscript that need to be clarified and/or justified.

 Below are specific comments as they relate to the reported study:

- It is difficult to understand the justification for including the mouse component of the work. The final sentence of the introduction is misleading. As it reads, it indicates that mice were used to demonstrate transmission (i.e. that mosquitoes actually fed on mice which were then monitored for evidence of infection). However, mice (along with mosquitoes) were used to indicate whether saliva collected using an in vitro method contained infectious virus and not to demonstrate transmission directly. If mice had been used to demonstrate transmission directly, then this would have added greatly to the novelty and significance of the study by demonstrating that transmission is affected in an animal model and not just using in vitro assessment of transmission (which is what almost all other studies do).
- The actual number of saliva samples tested was relatively low, so interpretation of the findings with regards to transmission blocking should be undertaken with caution, especially when extrapolating to the field.
- “Pluripotency” is not really an appropriate descriptive term in this context.
- The background in the abstract needs to mention that there is an efficacious vaccine and then state issues with its widespread roll-out (i.e. supply, logistics etc.). This provides a segue into why *Wolbachia* may be appropriate for YFV control.
- Also in the abstract (5 ^th^ line): *Aedes aegypti* is still the main (urban) vector, not “used to be”.
- Introduction: In lines 3 and 4 of paragraph 2, it is important to emphasise that *Haemagogus *and *Sabethes* are important sylvan vectors in South America. Africa has a different suite of sylvan vectors. Also, insert a comma after *Sabethes*, as the way it currently reads, it sounds as though they are all urban vectors.
- Also in this paragraph (lines 9-12), state that there was (thankfully) no evidence of urban transmission during these outbreaks.
- Line 10 of paragraph 3: Need a segue into the *Wolbachia*-based control. Something like “ *Wolbachia* is being deployed to limit arbovirus transmission…”. Then describe what *Wolbachia* actually is.
- Methods: Need to be consistent on whether it is PCR or RT PCR.
- Methods: Please provide references for the IFA and qRT-PCR in regard to the virus isolation.
- Methods: In the first paragraph of the mosquito saliva transmission assays (line 9), state whether saliva was from individual mosquitoes or from pools of saliva.
- Methods: Viral detection on (in) infected mosquitoes and mice:
  - Remove “infected” from this heading, as it is not known whether mosquitoes are infected until they are tested.
  - Also, line 1 of first paragraph: qPCR detects viral RNA.
  - What was the justification for using inoculation of mosquitoes and mice to demonstrate transmission? Why not a cell culture based system?
  - In terms of the mice, is there evidence that the strain used was highly susceptible to YFV infection? Why was this strain of mice used?
- In terms of the multiplex assay, can some data be supplied regarding the relative efficiencies of each of the components of the assay? How does sensitivity compare to singleplex assays? What were the limits of detection, especially for detection of YFV?
- More information on the quantification of *Wolbachia* and YFV would be beneficial, especially with respect to the RPS 17 sequence and why it was included.
- Results: The authors state that there “was no significant difference between infection rates resulting from the human or primate virus isolates.”. Please provide the statistical test used to compare rates and provide significance levels. Please provide statistical tests to show that infection rates were indeed significantly different between *Wolbachia*-positive and -negative mosquitoes.
- Results: Paragraph 2, lines 9-11 – this is speculation or if there is previously published work on this it should be provided – in the discussion.
- Figure 3:
  - Were only the head and thorax of YFV-infected mosquitoes tested for *Wolbachia* density? One would think not, given they are *Wolbachia* infected. Clarify.
  - Line 4, the sentence staring with (B) needs to state that these were the YFV copy numbers from the start.
  - In the last sentence, what does the significance level refer to?
  - Was this absolute or relative quantification of YFV RNA? Clarify.
- Results: The number of saliva samples from WT and wMel+ was 8 for each of the viruses (primate and human). Why was such a relatively low number (especially of the WT) of saliva samples tested? In Figure 3, there was a much larger number of mosquitoes tested. Was transmission not attempted with these mosquitoes? The number of saliva samples tested really was very low compared to what could and should have been tested (especially by mosquito injection, where there shouldn’t really be any limitation to the number of samples processed).
- Figure 4: The legend and figures are the wrong way around. Do Figures 4A and 4C actually refer to wMel+ infected saliva?
- The discussion needs to consider some important factors:
  - What are the possible mechanisms causing the *Wolbachia*-mediated virus inhibition? This is pertinent in this case, because the effect of subsequent blood meals was examined.
  - *Wolbachia*-based control strategies are undoubtedly promising tools for control of *Aedes aegypti* transmitted viruses. However, the authors should discuss any potential issues that could arise with *Wolbachia*-based approaches in the future.

I have read this submission. I believe that I have an appropriate level of expertise to confirm that it is of an acceptable scientific standard, however I have significant reservations, as outlined above.

# Response

**Published:** 2019-04-08

**Authors:** Luciano Moreira

**Affiliations:** FIOCRUZ, Brazil

Reviewer – Dr. Andrew van den Hurk

The deployment of *Aedes aegypti *transinfected with the *Wolbachia* is a highly promising strategy for suppressing the transmission of a number of globally important arboviruses, such as dengue, Zika, chikungunya and yellow fever viruses. In the current manuscript, the authors conducted laboratory-based experiments to assess the ability of the *w*Mel strain of *Wolbachia* to inhibit the transmission potential of two outbreak strains of yellow fever virus by *Aedes aegypti*. As has been demonstrated with other flaviviruses in numerous similar studies, *w*Mel inhibited replication and subsequent transmission when compared with *Wolbachia*-negative mosquitoes. This is a relatively well written manuscript describing experiments with outcomes of interest to readers of Gates Open Research. However, there are some components of the manuscript that need to be clarified and/or justified.

Below are specific comments as they relate to the reported study:

- It is difficult to understand the justification for including the mouse component of the work. The final sentence of the introduction is misleading. As it reads, it indicates that mice were used to demonstrate transmission (i.e. that mosquitoes actually fed on mice which were then monitored for evidence of infection). However, mice (along with mosquitoes) were used to indicate whether saliva collected using an in vitro method contained infectious virus and not to demonstrate transmission directly. If mice had been used to demonstrate transmission directly, then this would have added greatly to the novelty and significance of the study by demonstrating that transmission is affected in an animal model and not just using in vitro assessment of transmission (which is what almost all other studies do).

*>> Thank you for pointing this out. We agree with this and we have edited the text accordingly. Now the end of the introduction reads: “We found that Wolbachia had a major impact on virus replication in mosquitoes, as well as reduced the potential of YFV transmission via saliva, as indirectly determined via mosquitoes or a mouse model”.*

*The reason why we have not performed the experiment by feeding infected mosquitoes directly on the mice was because we had no biosafety approval to perform these experiments. Therefore, the saliva samples had to be transported to another institution (UFMG), where the mice were located, and then used there.*

 

- The actual number of saliva samples tested was relatively low, so interpretation of the findings with regards to transmission blocking should be undertaken with caution, especially when extrapolating to the field.

*>> We agree with the reviewer, but we think we were able to have good sampling numbers if we consider that 2 isolates (primate and human) were used and the results are quite similar.*

- “Pluripotency” is not really an appropriate descriptive term in this context.

*>> The idea of using the word “Pluripotency” was to describe the ability of Wolbachia to reduce transmission of several different pathogens. If there is no big issue on this, we would prefer to keep it and change Arbovirus to Arboviruses to better illustrate what we wanted.*

 

- The background in the abstract needs to mention that there is an efficacious vaccine and then state issues with its widespread roll-out (i.e. supply, logistics etc.). This provides a segue into why *Wolbachia* may be appropriate for YFV control.

*>> Thank you for pointing this out. We agree with the reviewer and edited the text accordingly to include this suggestion:*

**Background**: Yellow fever outbreaks have re-emerged in Brazil during 2016-18, with mortality rates up to 30%. Although urban transmission has not been reported since 1942, the risk of re-urbanization of yellow fever is significant, as *Aedes aegypti* is present in most tropical and sub-tropical cities in the World and used to be the main vector in the past. Although the YFV vaccine is safe and effective, it does not always reach populations at greatest risk of infection and there is an acknowledged global shortage of vaccine supply. The introgression of *Wolbachia* bacteria into *Ae. aegypti* mosquito populations is being trialed in several countries ( www.worldmosquito.org) as a biocontrol method against dengue, Zika and chikungunya. Here, we studied the ability of *Wolbachia* to reduce the transmission potential of *Ae. aegypti* mosquitoes for yellow fever virus (YFV).

 

- Also in the abstract (5 ^th^ line): *Aedes aegypti* is still the main (urban) vector, not “used to be”.

*>> OK. This has been corrected:*

Although urban transmission has not been reported since 1942, the risk of re-urbanization of yellow fever is significant, as *Aedes aegypti* is present in most tropical and sub-tropical cities in the World and still remains the main vector of urban YFV.

- Introduction: In lines 3 and 4 of paragraph 2, it is important to emphasise that *Haemagogus *and *Sabethes* are important sylvan vectors in South America. Africa has a different suite of sylvan vectors. Also, insert a comma after *Sabethes*, as the way it currently reads, it sounds as though they are all urban vectors.

*>> The text has been modified to accommodate the reviewer’s suggestion.*

The yellow fever virus (YFV) is a member of the Flaviviridae family and transmitted by sylvan mosquitoes of the genus *Haemagogus* and *Sabethes,* in South America and *Aedes aegypti* in urban settings ^5^^– ^^8^ .

- Also in this paragraph (lines 9-12), state that there was (thankfully) no evidence of urban transmission during these outbreaks.

*>> The text has been modified:*

“Although the last reported cases of urban transmission in Brazil occurred in 1942, in 2016–2017, the country faced major outbreaks of the disease mainly in the states of Minas Gerais, Espírito Santo and Rio de Janeiro. In 2018, the epidemic also extended to São Paulo State ^9^ . According to the Brazilian Ministry of Health, from July 2017 to April 2018, there were 1,127 YFV cases with 328 deaths, with no evidence of urban transmission.”

 

- Line 10 of paragraph 3:Need a segue into the “Wolbachia-based control. Something like “Wolbachia is being deployed to limit arbovirus transmission…”. Then describe what Wolbachia actually is.

*>> The text has been modified to accommodate the reviewer’s suggestion:*

“Population control of *Ae. aegypti* mosquitoes using insecticides has been a mainstay of vector-borne disease control methods for decades but is undermined by widespread insecticide resistance. A promising innovative strategy, based on a bacterium called *Wolbachia pipientis*, has been trialed in many countries. *Wolbachia *is a maternally transmitted bacterial endosymbiont and is naturally present in at least 40% of all insect species ^14^ .”

 

- Methods: Need to be consistent on whether it is PCR or RT PCR.

*>> For all the experiments related to the virus we used RT-qPCR. Regular PCR was used for the sequencing only. Some corrections were made on RT-qPCR throughout the text.*

 

- Methods: Please provide references for the IFA and qRT-PCR in regard to the virus isolation.

*>> Ok. Two references have been added.*

IFA was performed with a monoclonal YFV antibody donated by Evandro Chagas Institute (Arbovirology and Hemorrhagic Fevers Section) and conjugated goat anti-mouse IgG labeled with fluorescein FITC (MP Biomedicals) according to Adungo *et al*. 2016 ^18^ with modifications. Images were obtained using an Olympus microscope model BX51 with DP72 camera and DP-2BSW software. Viral molecular confirmation was performed using RNA extracted from the culture supernatant of each isolate, followed by amplification of the genetic material as described below in the viral detection section according to Domingo *et al*. 2012 ^19^. For mosquito infections, the YFV isolates were replicated in C636 cells ( *Ae. albopictus*) cultured in Leibovitz 15 medium (Gibco) supplemented with 10% fetal bovine serum (FBS) (Gibco) for 5 days at 28°C. Viral load was confirmed by RT-qPCR and later through plaque assays (PFU) in VERO cells (CCL81) grown in DMEM medium (Gibco) and 3% Carboxymethylcellulose (Sigma) supplemented with 2% FBS (Gibco) at 37°C and 5% CO _2_^20^ .

 

- Methods: In the first paragraph of the mosquito saliva transmission assays (line 9), state whether saliva was from individual mosquitoes or from pools of saliva.

*>> Saliva samples came from individual mosquitoes. The text has been modified for clarity.*

“Individual saliva samples were injected into WT mosquitoes, after 2 to 4 days of emergence.”

- Methods: Viral detection on (in) infected mosquitoes and mice:
  - Remove “infected” from this heading, as it is not known whether mosquitoes are infected until they are tested.

*>> Ok, done:*

“Viral detection in mosquitoes and mice”

 

- Also, line 1 of first paragraph: qPCR detects viral RNA.

*>> Ok, done:*

“Detection of viral RNA on infected mosquitoes and mice samples were performed through quantitative real-time PCR (RT-qPCR) using LightCycler® Multiplex RNA Virus Master (Roche), according to the previously published protocol.”

 

- What was the justification for using inoculation of mosquitoes and mice to demonstrate transmission? Why not a cell culture based system?

*>> We have tested, in the past, individual saliva samples into cell culture and we were not able to have successful viral growth. Therefore, we have chosen to use these indirect methods (mosquito or mice) to show that the virus was indeed infectious. If the mosquito or mouse became infected it is a good sign that the saliva contained active and infectious virus.*

- In terms of the mice, is there evidence that the strain used was highly susceptible to YFV infection? Why was this strain of mice used?

*>> In this study A129-/-SV129 strain of mice was used. The A129-/- mice strain was chosen based on the fact that they are deficient in important innate immune components, more specifically the type I interferons* *a* */* *b* *receptor. Type I Interferons ( IFN-α/β) plays a significant role in preventing viral replication and protecting against arboviral infections such as Zika, Dengue and Yellow fever viruses (1-5). They are the gold standard models to evaluate virus replication and therapeutical drugs due their elevated susceptibility to infection.*

*Lazear HM, Govero J, Smith AM, Platt DJ, Fernandez E, Miner JJ, Diamond MS. (2016). A mouse model of Zika virus pathogenesis. Cell Host Microbe, 19, 1-11. Published online April 5, 2016.* *http://dx.doi.org/10.1016/j.chom.2016.03.010*

*Rossi SL, Tesh RB, Azar SR, Muruato AE, Hanley KA, Auguste AJ, Langsjoen RM, Paessler S, Basilaski N, Weaver SC. (2016). Characterization of a novel murine model to study zika virus. Am J Trop Med Hyg, 16-0111; Published online March 28, 2016.* *http://dx.doi.org/10.4269/ajtmh.16-0111*

*Sarathy* *VV,* *Milligan* *GN,* *Bourne* *N,* *Barrett* *ADT. (2015). Mouse models of dengue virus infection for vaccine testing.* *Vaccine. 2015 Dec 10; 33(50): 7051–7060.* *10.1016/j.vaccine.2015.09.112* *.*

*Yauch LE* *,* *Shresta S* *. (2008). Mouse models of dengue virus infection and disease.* *Antiviral Res.* *2008 Nov;80(2):87-93. doi: 10.1016/j.antiviral.2008.06.010.*

*Meier KC* *1,* *Gardner CL* *,* *Khoretonenko MV* *,* *Klimstra WB* *,* *Ryman KD* *. (2009). A mouse model for studying viscerotropic disease caused by yellow fever virus infection.* *PLoS Pathog.* *2009 Oct;5(10):e1000614. doi: 10.1371/journal.ppat.1000614*

 

- In terms of the multiplex assay, can some data be supplied regarding the relative efficiencies of each of the components of the assay? How does sensitivity compare to singleplex assays? What were the limits of detection, especially for detection of YFV?

*>> We have done, please see the graph and table linked here, a comparison of the single or multiplex assay with a plasmid standard curve and the results are quite similar.*

 

- More information on the quantification of *Wolbachia* and YFV would be beneficial, especially with respect to the RPS 17 sequence and why it was included.

*>> Ok, done:*

A fraction (1/20) of the total isolated RNA was used in the reactions. Head and thorax samples from YFV-challenged mosquitoes were analyzed in duplicate through RT-qPCR and viral and *Wolbachia* quantification were performed in comparison with serial dilution of a standard curve of the respective genes cloned into the pGEMT plasmid (Promega) ^16,27^. Therefore, it was possible to calculate the number of copies per tissue. As a mosquito control gene we used the RPS 17S sequence of *Ae. aegypti* (Moreira 2009) ^15^.

 

- Results: The authors state that there “was no significant difference between infection rates resulting from the human or primate virus isolates.”. Please provide the statistical test used to compare rates and provide significance levels. Please provide statistical tests to show that infection rates were indeed significantly different between *Wolbachia*-positive and -negative mosquitoes.

*>> We have included the following statement: There was no significant difference between infection rates resulting from the human or primate virus isolates (Mann-Whitney U test p>0.05). As for the comparisons between different groups we have added the corresponding statistical analyses values in the legend of Fig 3.*

Red lines indicate the median *w*Mel copies (Mann-Whitney U test, ** *p*=0.0062). ( **B**) Analysis of copies of viral RNA on 7dpf -WT x *w*Mel+ (** *p*=0.0028) and YFV Human x Primate WT ( *p* = 0.43), ( **C**) 14dpf YFV Human and Primate WT x *w*Mel + (**** *p*<0.0001), YFV Human x Primate WT ( *p*=0.75), YFV Human x Primate extra blood meal WT ( *p*=0.78), YFV Human WT x WT extra blood meal (** *p*=0.0061) and YFV Primate WT x WT extra blood meal (** *p*= 0.0056) and ( **D**) 21 dpf -WT x *w*Mel+ (**** *p*=0.0001) and YFV Human x Primate ( *p*= 0.51). Empty black circles and triangles are WT mosquitoes, whereas empty green circles and triangles depict mosquitoes with *w*Mel +. Black filled circles and triangles are mosquitoes that received a second blood meal. The red line indicates the median YFV copies.

Wild type (WT) or positive ( *w*Mel +) were orally infected with two YFV isolates and virus dissemination in mosquitoes was analyzed at different times post infection. ( **A**) YFV infected mosquitoes’ heads and thoraces were analyzed for *Wolbachia* density at different times post-infection through real time RT-qPCR, based on a *Wolbachia* standard curve. Red lines indicate the median *w*Mel copies (Mann-Whitney U test, ** *p*=0.0062). ( **B**) Analysis of copies of viral RNA on 7dpf -WT x *w*Mel+ (** *p*=0.0028) and YFV Human x Primate WT ( *p* = 0.43), ( **C**) 14dpf YFV Human and Primate WT x *w*Mel + (**** *p*<0.0001), YFV Human x Primate WT ( *p*=0.75), YFV Human x Primate extra blood meal WT ( *p*=0.78), YFV Human WT x WT extra blood meal (** *p*=0.0061) and YFV Primate WT x WT extra blood meal (** *p*= 0.0056) and ( **D**) 21 dpf -WT x *w*Mel+ (**** *p*=0.0001) and YFV Human x Primate ( *p*= 0.51). Empty black circles and triangles are WT mosquitoes, whereas empty green circles and triangles depict mosquitoes with *w*Mel +. Black filled circles and triangles are mosquitoes that received a second blood meal. The red line indicates the median YFV copies.

 

- Results: Paragraph 2, lines 9-11 – this is speculation or if there is previously published work on this it should be provided – in the discussion.

*>> We apologize but we are not sure where this is on the text. Could you please add more information on where you affirming there is a speculation? We could not find where you mentioned about Paragraph 2, lines 9-11.*

 Figure 3:

- Were only the head and thorax of YFV-infected mosquitoes tested for *Wolbachia* density? One would think not, given they are *Wolbachia* infected. Clarify.

*>> The same samples that were used to evaluate the infection were also tested for Wolbachia quantification in the multiplex assay. The analysis of the results was done by targeting the gene of interest. In this way, it generated the graph 3A for the quantification of Wolbachia in the analyzed tissue that was head and thorax. As Wolbachia is present in practically all mosquito tissues, these analyses are possible.*

 

- Line 4, the sentence staring with (B) needs to state that these were the YFV copy numbers from the start.

*>> Ok, done:*

(B) Analysis of copies of viral RNA on 7dpf -WT x wMel+ (** p=0.0028) and YFV Human x Primate WT (p = 0.43)(...)

- In the last sentence, what does the significance level refer to?

*>> the level of significance was added for all pairwise comparisons in the legend text.*

- Was this absolute or relative quantification of YFV RNA? Clarify.

*>> as stated on the legend title both the Wolbachia as well as the YFV were done via absolute quantification.*

- Results: The number of saliva samples from WT and wMel+ was 8 for each of the viruses (primate and human). Why was such a relatively low number (especially of the WT) of saliva samples tested? In Figure 3, there was a much larger number of mosquitoes tested. Was transmission not attempted with these mosquitoes? The number of saliva samples tested really was very low compared to what could and should have been tested (especially by mosquito injection, where there shouldn’t really be any limitation to the number of samples processed).

*>> Each saliva sample was collected and processed individually. Each saliva sample was injected into up to 8 naïve mosquitoes. This clearly show how sample numbers are multiplied (up to 64 for each group). It was merely the question of numbers and trying to lower the costs.*

*We have collected 20 saliva samples from each group. Eight were used to inject the mosquitoes (as explained above) and the other 12 were used in two (pooled) to inject mice (6 mice per group).*

- Figure 4: The legend and figures are the wrong way around. Do Figures 4A and 4C actually refer to wMel+ infected saliva?

*>> Thank you for pointing this out! The text has been corrected:*

Saliva from both groups of infected mosquitoes were collected at 14 dpf. Individual saliva samples (WT or *w*Mel +) were injected into eight naïve (WT) mosquitoes (bars) and, after five days, these injected mosquitoes were analyzed. ( **A**) Mosquitoes injected with WT mosquito saliva or ( **B**) *w*Mel+ mosquitoes, challenged with human virus. ( **C**) Mosquitoes injected with WT mosquito saliva or ( **D**) *w*Mel+ mosquitoes, challenged with primate virus. Values below each bar depicts the viral load of each mosquito head and thorax which donated that saliva. Positive mosquitoes were quantified through RT-qPCR and the grey-scale represents the number of YFV copies (0 to 10 ^6^ copies), per mosquito.

 

- The discussion needs to consider some important factors:
  - What are the possible mechanisms causing the *Wolbachia*-mediated virus inhibition? This is pertinent in this case, because the effect of subsequent blood meals was examined.

*>> In paragraph 5 of the discussion we include this information:*

Another interesting fact of this work was the increase in viral load observed after the second blood feeding in WT mosquitoes. This same fact was not observed in *w*Mel + mosquitoes. This shows that the blocking ability of *Wolbachia* persists even after the addition of extra blood nutrients (through a second blood meal) and that its blocking effect occurs within 7 days after infection. The reason to include the second blood meal was that antibodies to YFV could be present in the blood and therefore, promote negative effect towards the virus in WT mosquitoes, but this was not the case. Caragata *et al*. (2013) ^45^ studied the effect of cholesterol towards the Drosophila C virus. This mechanism could be present in our experimental mosquitoes, but further studies on this aspect should be developed.

 

- *Wolbachia*-based control strategies are undoubtedly promising tools for control of *Aedes aegypti* transmitted viruses. However, the authors should discuss any potential issues that could arise with *Wolbachia*-based approaches in the future.

*>> It is important to consider the possible vector competence of other mosquito species and the possibility of Wolbachia/ virus evolution and lack of interference in this system. If that is the case, other strategies should be consider, as the use of other strains of Wolbachia to try to block virus transmission by that particular mosquito species. We have added this to the end of the discussion. “Lastly, it is important to consider the possible vector competence of other mosquito species and the possibility of Wolbachia/ virus evolution and lack of interference in this system. If that is the case, other strategies should be consider, as the use of other strains of Wolbachia to try to block virus transmission by that particular mosquito species. Integration of complementary strategies are the best solution for arbovirus control.”*
